# A system dynamics-based model for the evolution of environmental group events

**DOI:** 10.1038/s41598-024-59283-1

**Published:** 2024-04-27

**Authors:** Xue-ting Qi, Fanliang Bu

**Affiliations:** People’s Public Security University of China, Beijing, China

**Keywords:** System dynamics, Environmental mass events, Game theory, Socioeconomic scenarios, Sustainability

## Abstract

Based on the system dynamics theory, this paper establishes an environmental mass event evolution model and explores the evolution law of mass events caused by environmental problems. From a methodological point of view, the mixed-strategy evolutionary game principle and dynamic punishment measures are combined, and simulation analysis is carried out by Anylogic software, and the results show that there is no stable evolutionary equilibrium solution for the two sides of the game in the traditional asymmetric mixed-strategy game model, and after adjusting the game payoff matrix and incorporating the dynamic punishment strategy, stable evolutionary equilibrium solutions appear in the evolutionary game model, and the system begins to tend to be stabilized. The process and conclusions of the simulation experiment provide methodological reference and theoretical support for the analysis of the evolution of environmental mass events.

## Introduction

Following the opening up and reform, China's inland region has witnessed the emergence of environmental challenges, as the pace of industrialization and urbanization accelerates. Regrettably, the lack of a well-established framework to address environmental issues has resulted in rising social contradictions and public disputes. The current trend of environmental mass incidents is characterized by four key features.

Firstly, the geographic distribution of these incidents has shifted from regional to national scale, encompassing China's eastern, central, and western regions. Secondly, the number of individuals involved in environmental mass incidents has been on the rise, with participants having both direct and indirect interests. Thirdly, the total number of environmental mass incidents has undergone an exponential increase, as evidenced by the number of letters and visits received by China's inland environmental protection system. Fourthly, environmental defenders have increasingly resorted to violent means to assert their environmental rights, with participants displaying extreme behaviors and violent resistance^1^.

The escalating number of environmental mass incidents in China's inland region has emerged as a critical threat to social stability. While existing academic research has predominantly focused on theoretical arguments about environmental problems and group games, there is a need for further analysis of the evolution process and the law of environmental mass incidents.

### Environmental issues and group gaming

The evolution of environmental mass events is essentially a group gaming process involving multiple parties. Game theory has been successfully applied in the field of environmental protection in scenarios such as climate change, ozone problems, international water resources problems, nuclear leakage pollution, etc. B. W. Kernighan et al^2^ first applied game theory to the analysis of cross-regional environmental pollution events, and then the game analysis methodology became a hot topic in the field of environmental protection. Tsai et al.^3–5^ compared and analyzed the applicability of repeated game payoff matrices and differential game payoff matrices in discrete-time and continuous-time problems, respectively, in a global environmental pollution model, and also proved the existence of nonlinear Markov equilibrium solutions in a first-order symmetric two-party game system. Zeeuw et al^6^ constructed an open-loop linear Markov noncooperative game model in the background of the acid rain problem of Europe and solved the corresponding problem with Nash equilibrium solutions. Mason et al^7^ further extended the model to higher-order asymmetric multi-party games based on the studies of Zeeuw et al. and Tsai et al. and obtained formal nonlinear Markov equilibrium solutions under the corresponding assumptions.

In recent years, there have also many scholars on the environmental pollution problem using traditional game theory to carry out evolutionary analysis and give the Nash equilibrium solution of the corresponding program^8–13^ . However, in the analysis process of these cases, it is presupposed that both sides of the game have complete rationality throughout the game, and in the evolution process of environmental mass events, both sides of the game with complete rationality are often inconsistent with reality. In the reality of environmental mass events in the game process are repeated for a long time, the two sides of the game through the other side of the strategy and the influence of external conditions continue to adjust their strategy, so some scholars^14^ also began to try to use the evolutionary game theory to carry out the analysis of environmental mass events, but the evolutionary game theory is only for the game equilibrium to do a preliminary analysis, did not carry out a more in-depth study of the evolutionary process.

### Environmental issues and group gaming

Evolutionary Game Theory is a theory that combines traditional game theory and dynamic evolutionary process, which was first proposed by Smith and Price et al^15^ when they studied asymmetric games, the theory no longer treats the game participants as completely rational research objects, but as finite rationality research objects, and game participants can gradually reach an evolutionary equilibrium state through The participants of the game can adjust their strategies through continuous trial and error and learning to gradually reach the equilibrium state of evolution, which has commonality with biological evolution, and the equilibrium reached refers to the function of the evolutionary process, and thus historical factors, institutional factors, and minor details of the evolutionary process will have an impact on the multiple equilibrium choices in the process of the game. Gale and Eaves et al^16^ further advanced the application of evolutionary game theory in the field of finite rational group decision-making based on Smith and Price et al. In the twenty-first century, with a series of results achieved by evolutionary game theory in the field of economics^17–21^, the scope of its application has gradually started from symmetric to asymmetric games, and at the same time, it has generated some new branches, one of which is the equilibrium strategy problem. One of them is the equilibrium strategy problem^22^.

Evolutionary games have two keys in the analysis of environmental mass events, namely, replicator dynamic equations and evolutionary equilibrium strategies. Replicator dynamic and evolutionary equilibrium strategy together constitute the most core basic concepts of evolutionary game theory, which respectively characterize the stable state of the evolutionary game and the dynamic convergence process to this stable state. The expansion of the concept of evolutionary equilibrium strategy and the dynamics of the evolutionary game theory constitute the main content of the development of evolutionary game theory.

Replication dynamics leads to rapid convergence of results by using the idea of imitative learning of dominant strategies during the evolutionary process so that the number of individuals in the population choosing the more successful strategy increases. Cressman^23^ applied replication dynamics to the evolutionary analysis of two biological populations to find a stable solution to the system which is the Pareto-optimal solution. Plank^24^ considered the equilibrium allocation of specific replicated dynamics of the game parties in the case of multiple participants. Sigalou et al^25^ analyzed the conditions under which the replicated dynamics are used to make the evolution converge to a stable structure in a continuous decision space.

Hirshleifer et al.^26^ argued that an evolutionary equilibrium means a dynamically stable equilibrium point that is locally and asymptotically stable such that from any state of the dynamical system, the evolution eventually converges to that equilibrium point. Friedman et al.^27^ argued that evolutionary equilibrium should be a broader concept and proposed that every Nash equilibrium solution in a dynamic system is an evolutionary equilibrium point. Meanwhile, evolutionary equilibrium and Nash equilibrium are not equivalent in dynamic systems, and the equilibrium strategy is not necessarily an evolutionary equilibrium. However, incorporating the replication dynamic mechanism can ensure that the equilibrium strategy is equivalent to the evolutionary equilibrium. Evolutionary games are still divided on the concept of equilibrium strategy, and the relationship between evolutionary equilibrium and equilibrium strategy needs to be further analyzed and defined in depth in the context of environmental mass events. System Dynamics is summarized based on game theory, to adapt to the management needs of modern social systems and developed. In the mid-1950s, the Massachusetts Institute of Technology (MIT) Professor J. W. Forrester founded the theory of system dynamics^28^, and then system dynamics in national defense^29^, social science^30^, medicine^31^, ecology^32^ and other fields have been widely studied and disseminated.

System dynamics allows decision-makers to construct dynamic models to simulate real-world problems based on previous historical experience and through feedback system theory so that system dynamics models can self-regulate through cyclic feedback. Meanwhile, using auxiliary tools such as system simulation software, the model can be calculated by simulating the evolution under different strategies at a lower cost in a shorter period.

Depending on the function, models are subdivided into analytical and simulation models, in analytical models the evolutionary results depend only on the model inputs, however, solutions for analytical models are not always available or may be difficult to find. In simulation models. A simulation model can be thought of as a set of rules (e.g., equations, flowcharts, state machines, meta-cellular automata) that define how the modeled system will change in the future given its current state. Simulation is the process of "executing" the model so that it changes state (discrete or continuous) over time, which will be modeled in this paper based on the characteristics of the evolution of environmental mass events.

In terms of simulation modeling, Kim^33^ constructed an initial mixed-strategy game model using a system dynamics model and made a qualitative analysis of the dynamic game system by simulating the game evolution process. Petia et al.^34^ constructed an oligopolistic two-party game model through system dynamics to describe the relationship between the two competitors, and the simulation results showed that after some parameters in the constructed model were adjusted, the dynamic system Hopf divergence occurred internally, which may either appear in a complex state such as alternating cycles or chaos or converge to a finite cycle with unconventional equilibrium.

This paper incorporates system dynamics in the process of the evolutionary game of environmental mass events and analyzes the evolutionary equilibrium of the asymmetric mixed-strategy dynamic game of environmental mass events under the uncertainty of parameter information.

## Evolutionary game analysis and modeling

By analyzing the relationship between environmental problems and mass events, this chapter constructs a set of environmental mass event evolution models based on system dynamics theory.

### A mixed-strategy evolutionary game between regulators and environmentally polluting firms

In the process of environmental governance, there is a game relationship between government regulatory authorities and environmental pollution enterprises. The essence of the game is the uncertainty and mutual influence that both parties face in decision-making, and environmentally polluting enterprises and government regulators face the same problem in formulating discharge strategies. There are two strategies for environmental polluting firms: to discharge the pollutants directly or to carry out pre-treatment before discharging them, and there are two strategies for government regulators: to sample and inspect or not to inspect.

From the perspective of environmentally polluting enterprises, regarding the treatment of pollutants, their direct discharge can save pre-treatment costs, but it will also increase the additional cost of pollution treatment by government regulators, whereas pre-treatment and then discharge will increase the cost of enterprises, but it can reduce the additional cost of pollution treatment by government regulators. This involves a choice between the economic interests of enterprises and their environmental responsibilities. At the same time, enterprises also need to pay a certain amount of money for accepting sampling inspections, thus affecting their decision-making and economic efficiency.

The decision-making of government regulators is also affected in many ways. Sample inspections can raise the environmental awareness and sense of responsibility of sewage enterprises and reduce the risk of environmental pollution. However, if government regulators do not carry out inspections, sewage enterprises will think that there is a lack of supervision, thus increasing the emission of pollutants and aggravating environmental pollution. In addition, in addition to the daily sewage treatment costs, government regulators will need to incur additional costs for post-treatment if the pollution situation is serious, thus increasing the cost burden on the government.

Therefore, the game problem between government regulators and environmental pollution enterprises needs to take into account various factors, including economic, environmental protection, legal, and other factors. Only by establishing an effective cooperation mechanism and coordination mechanism between government regulators and environmental pollution enterprises can the goal of environmental governance be realized and the goal of sustainable development be achieved. The simplified game benefit matrix between government regulators and environmental pollution enterprises is represented as shown in the Table 1.

**Table 1:** Game payoff matrix between government administration and environmental pollution firms.

Environmental pollution business strategy	Government regulator strategy	
	Sampling (ν)	Non-sampling
Direct emissions (μ)	$A_{1} ,A_{2}$	$B_{1} ,B_{2}$
Pretreatment emissions	$C_{1} ,C_{2}$	$D_{1} ,D_{2}$

For a sewage-producing enterprise, the optimal strategy is to pre-treat the discharge of pollutants when the government administration carries out sampling inspections, i.e., $C_{1} > A_{1}$; and when the government administration does not carry out inspections, direct discharge is the optimal strategy for the enterprise, i.e., B. The optimal strategy for the enterprise is to discharge pollutants directly, i.e., $B_{1} > D_{1}$.

For governmental authorities, the best strategy is to conduct sampling inspections when enterprises discharge pollutants directly without any treatment, i.e., $A_{2} > B_{2}$; and the best strategy is not to conduct sampling inspections when enterprises have pre-treated their discharges, i.e., $D_{2} > C_{2}$.

Assume that the mixed strategy of the environmental pollution enterprise is $\varepsilon_{c} = \left( {u,1 - u} \right)$ and $v$ is the probability that the regulator adopts the sampling inspection strategy. The expected utility functions of the environmental pollution enterprise and the government regulator are $\eta_{c} \left( {\varepsilon_{c} ,\varepsilon_{g} } \right)$ and $\eta_{g} \left( {\varepsilon_{c} ,\varepsilon_{g} } \right)$ respectively formally defined as follows:

1 $$\eta_{c} \left( {\varepsilon_{c} ,\varepsilon_{g} } \right) = u\left( {vA_{1} + (1 - v)B_{1} } \right) + \left( {1 - u} \right)\left( {vC_{1} + (1 - v)D_{1} } \right)$$

2 $$\eta_{g} \left( {\varepsilon_{c} ,\varepsilon_{g} } \right) = v\left( {uA_{2} + (1 - u)C_{2} } \right) + \left( {1 - v} \right)\left( {uB_{2} + (1 - u)D_{2} } \right)$$

Based on the maximization of revenue, the Nash equilibrium solution between the environmental polluting firms and the government regulator is found to be $\left( {\frac{{D_{2} - C_{2} }}{{A_{2} - B_{2} - C_{2} + D_{2} }},\frac{{B_{1} - D_{1} }}{{C_{1} - A_{1} + B_{1} - D_{1} }}} \right)$.

According to the idea of biological evolutionary replication dynamics, players in the game who choose lower-return strategies gradually change their decisions to learn from their opponents with higher-return strategies. In the example of polluting firms, the rate of change in the dynamics of the proportion of firms adopting a direct emission strategy and government regulators adopting sampling inspections can be represented by the replication dynamics equation:

3 $$\frac{du}{{dt}} = u\left( {\varphi_{d} - \varphi } \right) = u(1 - u)(\varphi_{d} - \varphi_{t} )$$

4 $$\frac{dv}{{dt}} = v\left( {\psi_{s} - \psi } \right) = v(1 - v)(\psi_{s} - \psi_{n} )$$

where the expected returns are the average returns under different strategies. For environmental polluters and government regulators, each expected return is shown in Tables 2 and 3, respectively:

**Table 2:** Representation of earnings expectations of environmentally polluting firms.

Expectation of benefits from direct sewage discharges by enterprises	Enterprise pretreatment emission benefit expectations	Average firm earnings expectations
$\varphi_{d}$	$\varphi_{t}$	$\varphi = u\varphi_{d} + \left( {1 - u} \right)\varphi_{t}$

**Table 3:** Government regulators' expectation of revenue expressed.

Expectations of regulatory gains in the government sector	Government departments do not regulate revenue expectations	Average earnings expectations of the government sector
$\psi_{s}$	$\psi_{n}$	$\psi = v\psi_{s} + \left( {1 - v} \right)\psi_{n}$

The game involves an interaction between environmental pollution enterprises and government regulators. The firms have the option to choose between direct discharge or pre-treatment discharge, while the regulator can opt for either sampling inspection or supplementary sampling inspection. The benefits of both parties are contingent upon the strategy choices made by the other. As a result, determining the expected benefits requires forecasting and evaluating the opposing party's approach while considering the risks and benefits associated with different strategies. Additionally, the game relationship between the two parties must also be factored into the equation.

For the mixed game, there is $$C_{1} > A_{1} ,B_{1} > D_{1} ,A_{2} > B_{2} ,D_{2} > C_{2}$$, such that $$F\left( u \right) = \frac{du}{{dt}},G\left( v \right) = \frac{dv}{{dt}}$$ Bringing $$\varphi_{d} = vA_{1} + \left( {1 - v} \right)B_{1} ,\varphi_{t} = vC_{1} + \left( {1 - v} \right)D_{1} ,$$$$\psi_{s} = uA_{2} + \left( {1 - u} \right)C_{2} ,\psi_{n} = uB_{2} + (1 - u)D_{2}$$ into Eqs. (3), and (4) we have:

5 $$F\left( u \right) = \frac{du}{{dt}} = u\left( {1 - u} \right)\left( {v(A_{1} - C_{1} ) + (1 - v)(B_{1} - D_{1} )} \right)$$

6 $$G\left( v \right) = \frac{dv}{{dt}} = v\left( {1 - v} \right)\left( {u(A_{2} - B_{2} ) + (1 - u)(C_{2} - D_{2} )} \right)$$

Noting that $C_{1} - A_{1} = A,B_{1} - D_{1} = B,A_{2} - B_{2} = C,D_{2} - C_{2} = D$, by the mixed game antecedent has $A,B,C,D > 0$, Eqs. (5) and (6) can be expressed as follows:

7 $$F\left( u \right) = \frac{du}{{dt}} = u\left( {1 - u} \right)\left( {(1 - v)B - vA} \right)$$

8 $$G\left( v \right) = \frac{dv}{{dt}} = v\left( {1 - v} \right)\left( {uC - (1 - u)D} \right)$$

Let $$X = \left( {\begin{array}{*{20}c} {F\left( u \right)} \\ {G\left( v \right)} \\ \end{array} } \right) = f(X,t) = 0$$, the equilibrium solution to the mixed game between the environmental pollution company and the government regulator has $$X_{1} = \left( {\begin{array}{*{20}c} 0 \\ 0 \\ \end{array} } \right),X_{2} = \left( {\begin{array}{*{20}c} 0 \\ 1 \\ \end{array} } \right),X_{3} = \left( {\begin{array}{*{20}c} 1 \\ 0 \\ \end{array} } \right),X_{4} = \left( {\begin{array}{*{20}c} 1 \\ 1 \\ \end{array} } \right),X_{5} = \left( {\begin{array}{*{20}c} {\frac{D}{C + D}} & {\frac{B}{A + B}} \\ \end{array} } \right)^{T}$$.

The Hessian matrix corresponding to $f(X,t)$ is solved to be an indeterminate matrix, none of $X(X_{1} ,X_{2} ,X_{3} ,X_{4} ,X_{5} )$ is an extreme point. In this system, all four points $X_{1} ,X_{2} ,X_{3} ,X_{4}$ are saddle points and $X_{5}$ is the center point. Therefore, there is no evolutionary stable equilibrium in this system, and the resultant oscillations do not converge, and even small changes may have a huge impact on the behavior of the system. In evolutionary games, the Nash equilibrium of a perfectly rational game is not necessarily an evolutionarily stable strategy.

### Mixed-strategy evolutionary games after incorporating a dynamic penalty mechanism

In the field of environmental governance, the establishment of penalties by government regulators is usually one of the most common strategies to prevent environmental polluters from exceeding emission standards. However, in the mixed-strategy game, increasing the punishment does not change the equilibrium point of the probability of violation of the punishment. Although the strategy can reduce the equilibrium point of the penalized in the short run, this conclusion ignores the fact that an increase in the intensity of the penalty affects the payment matrices of both sides of the game. Therefore, the government may need to develop a more elaborate environmental governance strategy.

Under these circumstances, government regulators may consider making flexible adjustments to the level of penalties for enterprises that directly discharge pollutants, so that they vary with the severity of environmental pollution. In this way, the government can more accurately intervene in environmental problems, and enterprises will be more motivated to comply with environmental laws and regulations. More importantly, the implementation of this strategy has an impact on the stability of the whole game model.

Therefore, when formulating environmental governance strategies, the government should consider the impact of different environmental governance measures on both sides of the game, to formulate more effective measures to protect the environment. In this way, the government can better safeguard the public interest and realize the coordinated development of the economy and environment.

Assuming that the degree of environmental disruption is positively and linearly correlated with the polluting enterprises, reflects the degree of environmental pollution; then, after incorporating the dynamic penalty mechanism, the expected return of the environmental polluting enterprises is changed from $A_{1}$ to $f_{{A_{1} }} \left( u \right) = A_{1} - u\theta$, where $\theta > 0$, brought into the Eqs. (5), (6) have:

9 $$F\left( u \right) = \frac{du}{{dt}} = u\left( {1 - u} \right)\left( {v(f_{u} - C_{1} ) + (1 - v)(B_{1} - D_{1} )} \right)$$

10 $$G\left( v \right) = \frac{dv}{{dt}} = v\left( {1 - v} \right)\left( {u(A_{2} - B_{2} ) + (1 - u)(C_{2} - D_{2} )} \right)$$

Let $g_{u} = C_{1} - f_{u} ,A = C_{1} - A_{1} ,B = B_{1} - D_{1} ,C = A_{2} - B_{2} ,D = D_{2} - C_{2}$, then Eqs. (9) and (10) can be expressed as:

11 $$F\left( u \right) = \frac{du}{{dt}} = u\left( {1 - u} \right)\left( {(1 - v)B - vg_{u} } \right)$$

12 $$G\left( v \right) = \frac{dv}{{dt}} = v\left( {1 - v} \right)\left( {uC - (1 - u)D} \right)$$

Let $$X = \left( {\begin{array}{*{20}c} {F\left( u \right)} \\ {G\left( v \right)} \\ \end{array} } \right) = f(X,t) = 0$$, find the equilibrium solution $$X_{1} = \left( {\begin{array}{*{20}c} 0 \\ 0 \\ \end{array} } \right),X_{2} = \left( {\begin{array}{*{20}c} 0 \\ 1 \\ \end{array} } \right),X_{3} = \left( {\begin{array}{*{20}c} 1 \\ 0 \\ \end{array} } \right),X_{4} = \left( {\begin{array}{*{20}c} 1 \\ 1 \\ \end{array} } \right),X_{5} = \left( {\begin{array}{*{20}c} {\frac{D}{C + D}} & {\frac{B}{{g_{u} + B}}} \\ \end{array} } \right)^{T}$$ of the mixed-strategy evolution game after incorporating the dynamic penalty mechanism. The corresponding Jacobian matrix is

$$J\left( X \right) = \frac{{\partial f\left( {X,t} \right)}}{\partial X} = \left[ {\begin{array}{*{20}c} {(1 - 2u)((1 - v)B - g_{u} v) - u(1 - u)vg_{u} ^{\prime}} & {u(u - 1)(g_{u} + B)} \\ {v(1 - v)(C + D)} & {(1 - 2v)(uC - (1 - u)D)} \\ \end{array} } \right]$$

When we bring in $\lambda E - J(X_{1} ) = 0$ and solve for the characteristic roots of $X_{1}$, we have $\lambda_{11} = B > 0,\lambda_{12} = - D < 0$, and determine that $X_{1}$ is a saddle point, and by the same reasoning, $X_{2} ,X_{3} ,X_{4}$ is a saddle point.

Bringing $$X_{5} = \left( {\begin{array}{*{20}c} {\frac{D}{C + D}} & {\frac{B}{{g_{u} + B}}} \\ \end{array} } \right)^{T}$$ into $J\left( X \right)$, there is:

13 $$J\left( {X_{5} } \right) = \frac{{\partial f\left( {X_{5} ,t} \right)}}{{\partial X_{5} }} = \left[ {\begin{array}{*{20}c} {\frac{{ - CBDg_{u} ^{\prime}}}{{(C + D)^{2} (B + g_{u} )}}} & {\frac{{ - \left( {g_{u} + B} \right)DC}}{{(C + D)^{2} }}} \\ {\frac{{B(C + D)g_{u} }}{{(B + g_{u} )^{2} }}} & 0 \\ \end{array} } \right]$$

Bringing in $\lambda E - J(X_{5} ) = 0$ , the characteristic equation is obtained:

14 $$\lambda^{2} + \frac{{BCDg_{u} ^{\prime}}}{{(C + D)^{2} (A + B + \frac{{Dg_{u} ^{\prime}}}{C + D})}}\lambda + \frac{{BCD(A + \frac{{Dg_{u} ^{\prime}}}{C + D})}}{{(C + D)(A + B + \frac{{Dg_{u} ^{\prime}}}{C + D})}} = 0$$

Find the characteristic root of $X_{5}$:

$$\lambda_{51} ,\lambda_{52} = - \frac{{BCDg_{u} ^{\prime}}}{{2(C + D)^{2} (A + B + \frac{{Dg_{u} ^{\prime}}}{C + D})}} \pm \frac{1}{2}\sqrt \Delta$$

included among these:

$$\Delta = \left( {\frac{{BCDg_{u} ^{\prime}}}{{(C + D)^{2} (A + B + \frac{{Dg_{u} ^{\prime}}}{C + D})}}} \right)^{2} - 4\frac{{BCD(A + \frac{{Dg_{u} ^{\prime}}}{C + D})}}{{(C + D)(A + B + \frac{{Dg_{u} ^{\prime}}}{C + D})}} < 0$$

Thus, the characteristic roots of $X_{5}$ are all negative real parts, the game is asymptotically stable, and the results converge.

## A game model for the evolution of group events in system dynamics environments

To study the influence of different mechanisms on the stability of the evolutionary equilibrium of environmental mass events, this section establishes a mixed-strategy evolutionary dynamics model to simulate the long-term dynamics of the two parties involved in the game to simulate the long-term dynamics of the behavioral trends of the game, to effectively fit the real environmental mass events.

**Figure 1:**
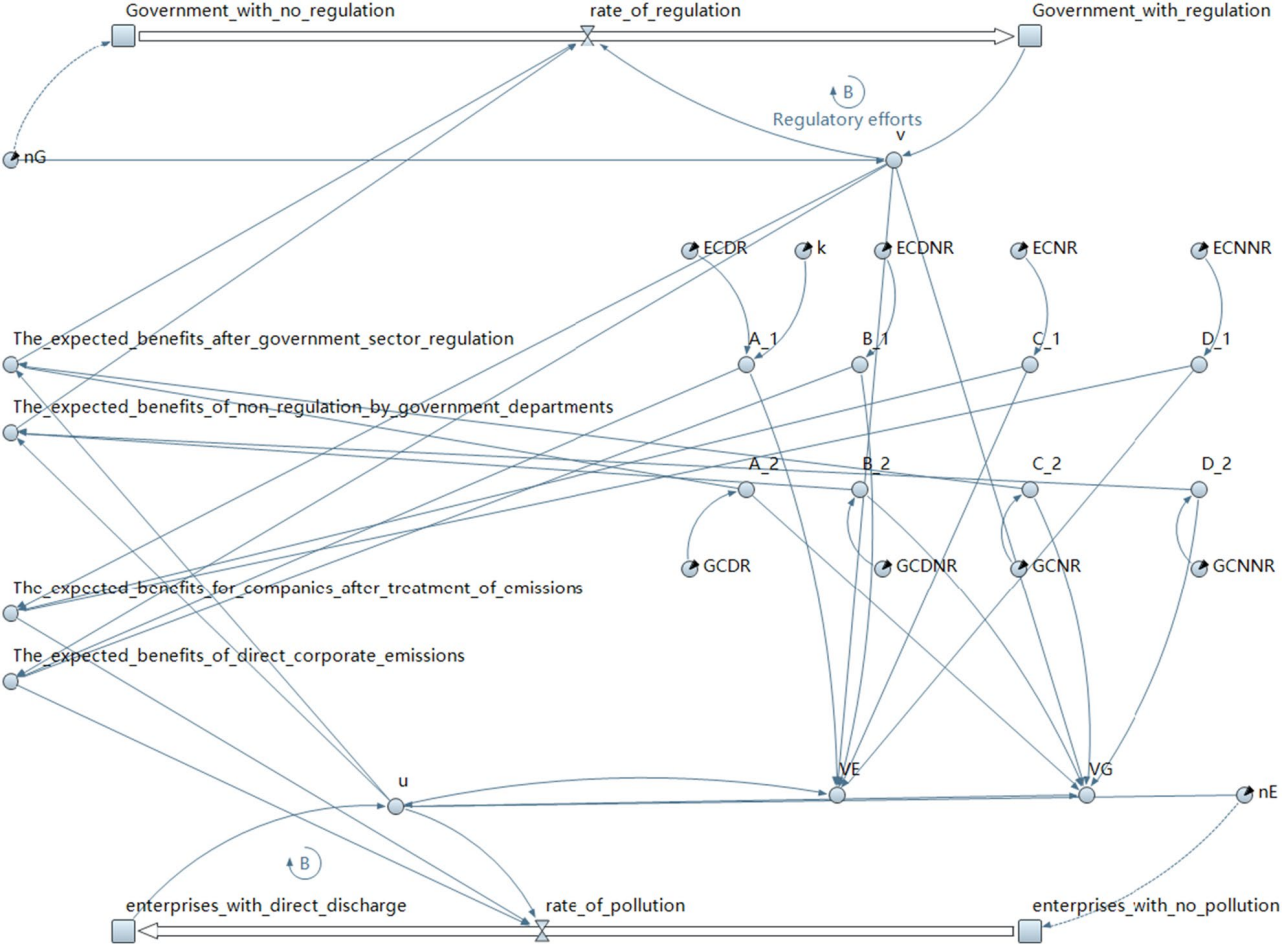
The SD model of the evolutionary game of environmental mass events.

AnyLogic simulation modeling software is used to construct an evolutionary game model between environmental pollution enterprises and government regulators using BASS diffusion as a prototype. As shown in Fig. 1:

This SD model for the evolution of the environmental mass event game consists of four streams, two flow rates, eight intermediate variables, and 12 external variables. Among them, the four streams describe the number of firms in the pollution production sector adopting the pretreatment discharge strategy and adopting the direct discharge strategy, as well as the proportion of personnel in the government regulatory sector adopting the monitoring strategy and adopting the no inspection strategy; the two streams are used to describe the rate of change of the personnel in the government regulatory sector adopting the inspection strategy and the firms adopting the direct discharge strategy.

In environmental mass events, the discharge strategy adopted by firms and the monitoring and inspection strategy adopted by personnel in the government regulator interacts with each other. This interaction is briefly analyzed in the previous two sections, to understand the evolution process and results under different strategies. The eight external variables correspond to Table 4:

**Table 4:** Description of external variables in the SD model.

Corresponding variable	SD model correspondence meaning	Initial value
$A_{1}$	ECDR (enterprise cost for direct discharge with regulation)	1
$A_{2}$	GCDR (government cost for direct discharge with regulation )	5
$B_{1}$	ECDNR(enterprise cost for direct discharge with no regulation)	5
$B_{2}$	GCDNR (government cost for direct discharge with no regulation)	2
$C_{1}$	ECNR(enterprise cost for no pollution with regulation)	4
$C_{2}$	GCNR (government cost for no pollution with regulation)	3
$D_{1}$	ECNNR (enterprise cost for no pollution with no regulation)	3
$D_{2}$	GCNNR (government cost for no pollution with no regulation)	4

**Figure 2:**
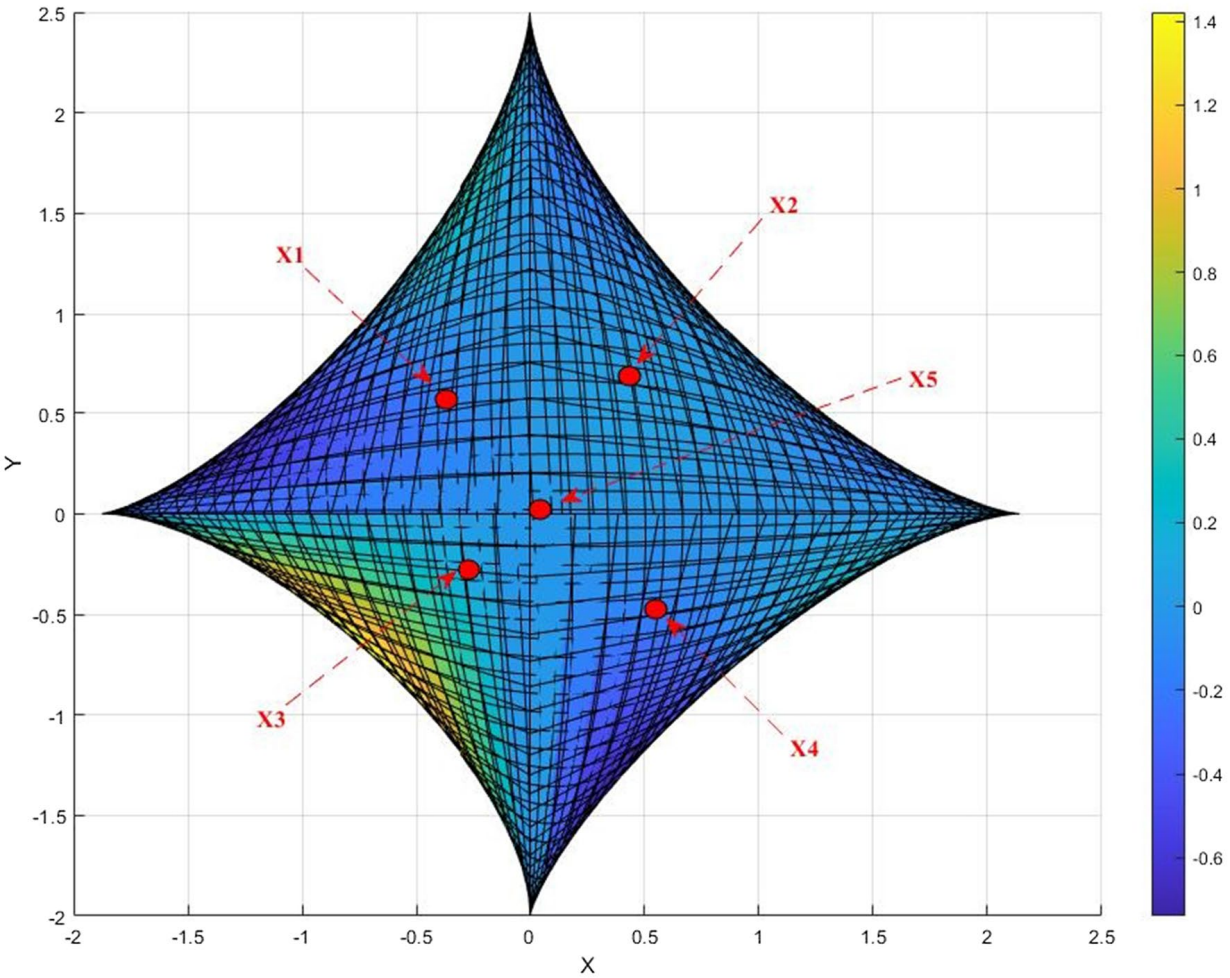
Graph of mixed game equilibrium function for the polluting firm and government regulator with dynamic penalties (the present system displays four saddle points on all points of $X_{1}$,$X_{2} ,X_{3} ,X_{4}$, while $X_{5}$ is an extreme point where the results are brought together. As a result, the system offers asymptotically stable equilibrium solutions. To achieve these stable solutions, the addition of dynamic penalties is required. After applying these penalties, the resulting oscillations converge to the equilibrium above solutions).

Figure 2 shows the equilibrium function of a mixed game between environmental polluting firms and government regulators, where dynamic penalties are used. This mathematical formula describes the optimal strategies of both parties in the game. In a mixed game, both sides have some information about each other's preferences and strategies, but not enough to make a fully informed decision.

The X-axis on the graph represents the strategy of the polluter, while the Y-axis represents the strategy of the government regulator. The function curves on the graph depict the game results of both sides under dynamic punishment measures. This graph can be used to analyze the incentives and outcomes of policy interventions or market conditions under pre-set dynamic punishment measures. It can also provide a reference for decision-making in related environmental mass events.

In summary, the equilibrium function plot of the mixed game between environmental polluters and government regulators concisely illustrates the complex economic and environmental interactions under dynamic penalties. This graph can inform policy decisions and promote a better understanding of this important issue. Figure 2 depicts the equilibrium function of a mixed game between environmental polluting firms and government regulators, in which dynamic penalties are implemented. This mathematical formula describes the optimal strategies of both parties in the game. A mixed game is a scenario where both sides possess some knowledge of each other's preferences and strategies, but lack enough information to make a fully informed decision.

In environmental mass incidents, penalizing enterprises with excessive emissions is one of the most common strategies used by government regulators. However, whether the implementation effect of increasing penalties meets expectations needs to be thoroughly studied from a game-theoretic perspective.

**Figure 3:**
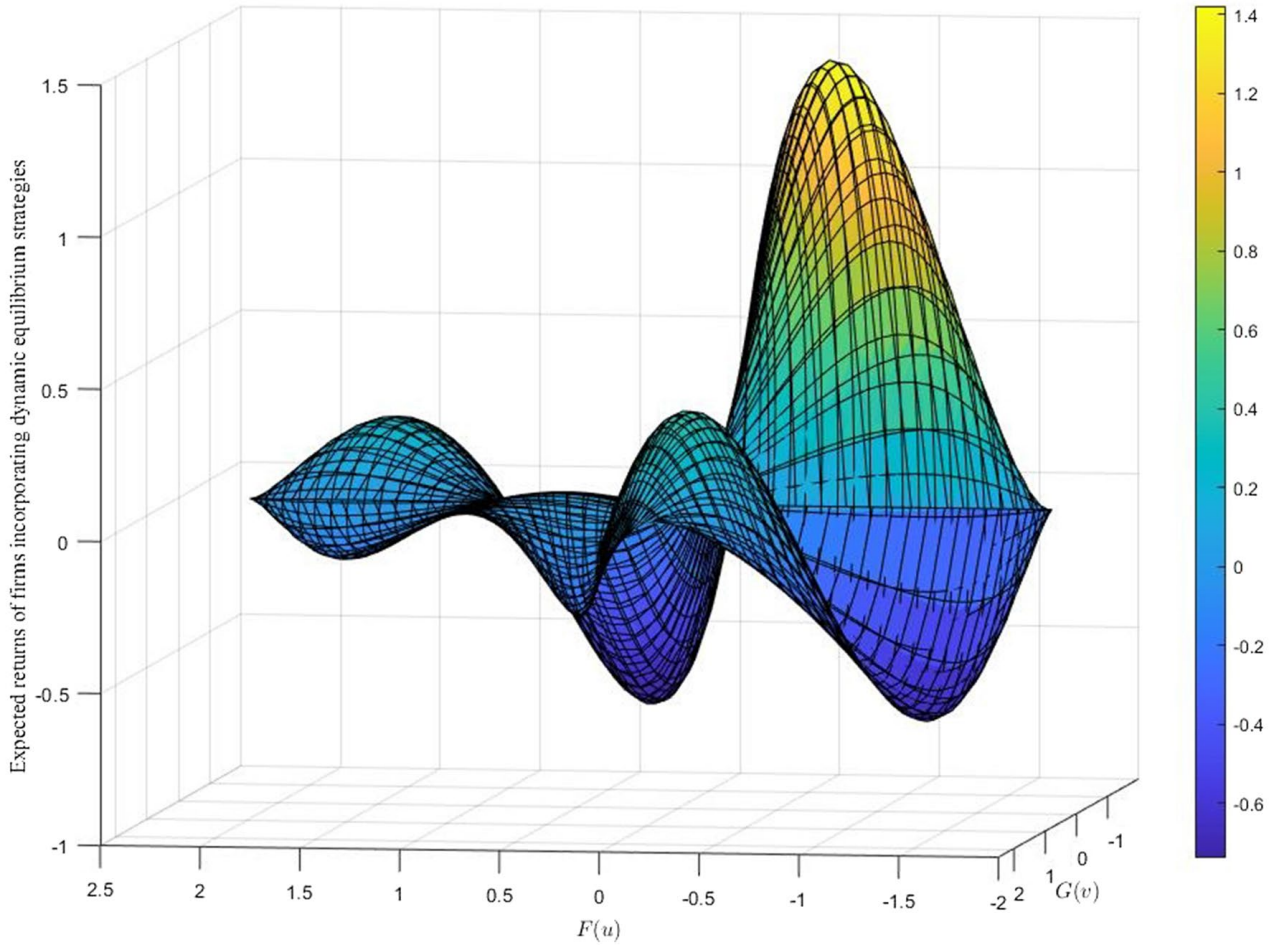
Graph of the company's return equilibrium function with changing penalties (the plot exhibits the saddle and extreme points that correspond to the initialization of the intermediate variables within the equilibrium function. This observation is essential for understanding the behavior of the system and its stability. It is worth noting that these intermediate variables play a significant role in the overall performance of the system).

Previous studies have shown that simply increasing penalties does not change the equilibrium of the probability of violating penalties^35,36^, which is a conclusion based on the mixed-strategy game. However, this conclusion ignores the actual impact of increasing penalties on the payoff matrices of both sides of the game. Please refer to Fig. 3 for a visual representation.If the government regulator can dynamically adjust the punishment for over-emitting enterprises according to the severity of environmental pollution, then the representation of the payoff matrix needs to be reconstructed to analyze the stability of the strategy more accurately after increasing the punishment.

Therefore, it is necessary to dynamically adjust the punishment strategy of the over-standard emission enterprises from the perspective of game theory and through in-depth analyses to better understand the limitations of the strategy and provide more effective measures for environmental governance.

## Conclusions and prospects

The application of an evolutionary game makes the limitations of the traditional game that presupposes the complete rationality of both sides of the game be overcome, combined with the system dynamics can be a better simulation of the environmental mass events, the dynamic characteristics of the equilibrium strategy can be examined from a global point of view, and the results of the analysis of the given item play an important role in the analysis of the evolution of the environmental mass events and the formulation of the corresponding decision-making.

The evolutionary analysis of environmental mass events is a complex work of multidisciplinary fields, this paper mainly uses the theory of evolutionary game and system dynamics to analyze and discuss the law of evolution under a series of preset conditions from the perspective of methodology. The data in the model, such as the revenue matrix, are only used as examples and do not involve the government and sewage enterprises in real environmental mass events. However, in the process of the system dynamic evolutionary game, the changes in the decision-making costs of the two sides of the game and the dynamic punishment mechanism on the evolution of the environmental mass events and the conclusions drawn have a certain reference value, at the same time, in this paper in the evolutionary analysis of the environmental mass events, the system dynamics of the system combined with the mixed-strategy evolutionary game, the use of simulation tools to intuitively compare the different strategies of different conditions of the dynamic game of the methodology on The method of using simulation tools to visually compare the dynamic game under different conditions of different strategies has certain reference significance for the analysis of the evolution of environmental mass events.

## Data Availability

All data generated or analysed during this study are included in this published article [and its supplementary information files].
